# Unified Parametric Optimization Framework for Microchannel Fin Geometries in High-Power Processor Cooling

**DOI:** 10.3390/mi17010086

**Published:** 2026-01-08

**Authors:** Abtin Ataei

**Affiliations:** Coherent Corporation, 375 Saxonburg Boulevard, Saxonburg, PA 16056, USA; abtin.ataei@coherent.com; Tel.: +1-(724)352-4455

**Keywords:** thermal resistance, pressure drop, continuation optimization, laminar–turbulent Nusselt blending, fin efficiency analysis

## Abstract

This study presents a unified parametric optimization framework for the thermal design of microchannel spreaders used in high-power processor cooling. The fin geometry is expressed in a shape-agnostic parametric form defined by fin thickness, top and bottom gap widths, and channel height, without prescribing a fixed cross-section. This approach accommodates practical fin profiles ranging from rectangular to tapered and V-shaped, allowing continuous geometric optimization within manufacturability and hydraulic limits. A coupled analytical–numerical model integrates conduction through the spreader base, interfacial resistance across the thermal interface material (TIM), and convection within the coolant channels while enforcing a pressure-drop constraint. The optimization uses a deterministic continuation method with smooth sigmoid mappings and penalty functions to maintain constraint satisfaction and stable convergence across the design space. The total thermal resistance ($R_{\mathrm{tot}}$) is minimized over spreader conductivities $k_{s}=400–2200$ W m^−1^ K^−1^ (copper to CVD diamond), inlet fluid velocities $U_{\mathrm{in}}=0.5–5.5$ m s^−1^, maximum pressure drops of 10–50 kPa, and fluid pass counts $N_{p}\in \{1,2,3\}$. The resulting maps of optimized fin dimensions as functions of $k_{s}$ provide continuous design charts that clarify how material conductivity, flow rate, and pass configuration collectively determine the geometry, minimizing total thermal resistance, thereby reducing chip temperature rise for a given heat load.

## 1. Introduction

Rising heat fluxes in high-performance processors have transformed liquid microchannel cooling from a research concept into an essential thermal management technology. The effectiveness of microchannels, which provide a large wetted perimeter relative to their footprint and very short conduction paths, was first demonstrated by Tuckerman and Pease, who achieved order-of-magnitude improvements in heat removal for VLSI chips [1,2]. Subsequent investigations have shown that channel geometry and flow hydraulics strongly influence convective resistance, while spreading and interfacial resistances within the solid stack often dominate near localized hot spots [3,4,5]. As a result, overall thermal performance depends not only on coolant flow but also on the thermophysical properties of the materials and the design of the interface layers. Enhancing the heat-spreader conductivity and minimizing thermal interface resistance can substantially lower junction temperatures for a given pumping power [6,7].

CVD diamond has emerged as a promising material for managing extreme heat fluxes because it combines ultra-high thermal conductivity with practical microfabrication compatibility and, in some integrations, direct bonding that can eliminate or significantly thin soft metal thermal interface layers [8]. At the same time, advances in channel architecture, including ribbed and wavy passages as well as tapered and converging fins, continue to highlight the trade-off between enhanced surface area and increased pressure drop [2,7,9]. The resulting design space is inherently multidimensional, as the most effective fin geometry depends jointly on the spreader conductivity, allowable hydraulic losses, flow regime, and manufacturability constraints [6,10,11].

Recent advances in microchannel heat sink research continue to highlight the strong interaction among channel geometry, hydraulic losses, and material selection under practical thermal loads. Studies on non-uniform and gradient fin structures demonstrate that geometric nonlinearity can significantly affect flow distribution, pressure drop, and convective performance [12,13]. Investigations of liquid-metal and hybrid-coolant microchannel systems further show how coolant selection and channel-width variation influence thermal resistance under restricted pumping power [14,15]. Optimization studies focused on complex cross-sections and constrained hydraulic budgets confirm the sensitivity of performance to fin shape and aspect ratio [16,17]. Recent experimental and numerical works on nested, bifurcated, and Tesla-patterned microchannel architectures demonstrate the benefits of multi-layer routing and engineered flow paths for enhancing uniformity and heat removal [18,19]. Collectively, these developments reinforce the importance of a unified and geometry-flexible optimization framework capable of capturing cross-sectional variations, material effects, and hydraulic constraints in a single formulation.

This work addresses these coupled dependencies by developing a unified, constraint-aware parametric framework that co-optimizes fin geometry across spreader conductivities ranging from copper to CVD diamond, inlet velocities, maximum pressure drops representative of practical pumps, and one to three fluid passes. The model integrates conduction through the spreader base, interfacial resistance across the thermal interface material (TIM), and convection within the coolant channels while enforcing geometric integrity, manufacturability bounds on feature sizes, and a system-level pressure-drop constraint. Heat transfer and hydraulic behavior are modeled using a blended laminar and turbulent Nusselt correlation together with a smooth Poiseuille number surrogate based on established theory, ensuring accurate transitions across Reynolds numbers [2,3,20,21]. A deterministic continuation strategy initializes the solver across material, architectural, and flow parameters, producing smooth and reproducible optimal geometry solutions while avoiding the oscillations that often appear in discrete, case-wise searches [7,22,23]. The analysis identifies conditions where fin height saturates below manufacturing limits, where asymmetric gaps become advantageous, and how increasing conductivity shifts the balance between spreading and convection, all within a framework that fully accounts for hydraulic penalties and fabrication constraints [5,9,10]. Collectively, the framework provides continuous, material-spanning guidance for designing advanced liquid-cooled processor packages while maintaining physical fidelity and practical relevance.

## 2. Numerical Methods

**Figure 1:**
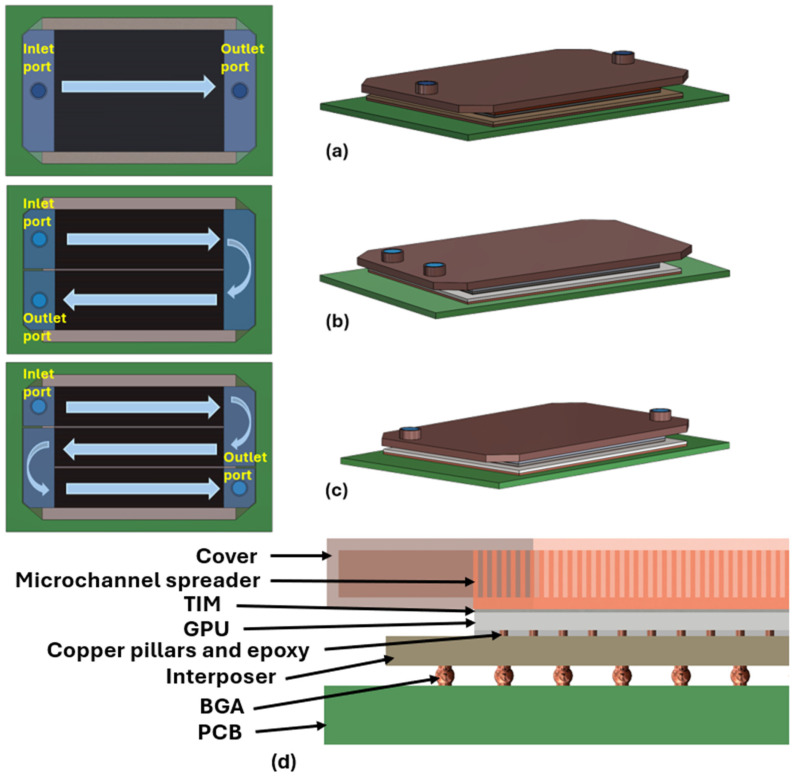
Microchannel cooling configurations and package structure. (**a**) Single pass, (**b**) two passes, and (**c**) three passes with indicated inlet and outlet ports. The arrows indicate the coolant flow direction through the microchannels. Different colors are used to distinguish the major package components and fluid domains. (**d**) Cross-sectional layout showing the cover, microchannel spreader, TIM, processor, copper pillars in epoxy, interposer, BGA, and PCB.

Figure 1 illustrates a model of the GPU cooling system under varied flow paths, which incorporates a microchannel spreader, a thermal interface material (TIM) layer between the GPU and the spreader, and a GPU connected to an interposer via copper pillars and an epoxy layer. The interposer is attached to a printed circuit board (PCB) through a ball grid array (BGA).

For clarity, representative package dimensions commonly used in GPU assemblies are noted here. Copper pillars typically have diameters of about 40 μm with a 40 μm copper height and a 20 μm solder cap, arranged on a 120 μm pitch with approximately 30 μm of underfill epoxy. The interposer thickness is generally around 0.5 mm, and BGA connections often use 0.5 mm solder balls on a 0.8 mm pitch arranged over a 40 × 40 grid. The PCB thickness is approximately 1.0 mm. These values are provided only as typical dimensions used in commercial packages and are not part of the optimization model, which focuses solely on the microchannel spreader geometry.

To develop a parametric optimization model for the fin geometry of a microchannel spreader, the governing equations must first be established. These equations form the foundation of the analytical framework, from which both the objective function and the associated constraints are derived. Once defined, the optimization model is solved to determine the optimum fin geometry as a function of the spreader material conductivity for various fluid inlet velocities, the number of passes and maximum pressure drops.

### 2.1. The Governing Equations

The temperature rise of the GPU (ΔT) under a heat load Q can be estimated using the total thermal resistance of the liquid cooling system ($R_{tot}$) as follows:

(1) $$∆T=QR_{tot}$$

The total thermal resistance ($R_{tot}$) is modeled as a series network of the TIM resistance ($R_{TIM}$), base conduction resistance ($R_{base}$), and convective resistance ($R_{conv}$) as given in Equation (2) [7].

(2) $$R_{tot}=R_{TIM}+R_{base}+R_{conv}$$

where the thermal resistance of the TIM layer ($R_{TIM}$) is calculated as a function of the thickness of the TIM layer ($t_{TIM}$), the conductivity of its material ($k_{TIM}$) and the cross-sectional area (A) as follows:

(3) $$R_{TIM}=\frac{t_{TIM}}{k_{TIM}A}$$

Similarly, the base conduction thermal resistance ($R_{base}$) is defined as Equation (4), where t is the thickness of the base and $k_{s}$ is the conductivity of the base material.

(4) $$\;R_{base}=\frac{t}{k_{s}A}$$

On the other hand, the convective thermal resistance ($R_{conv}$) heavily depends on the fins’ geometry and efficiency (η), in addition to the convective heat transfer coefficient or the film coefficient ($h_{c})$ as given in Equation (5) [7].

(5) $$R_{conv}=\frac{1}{h_{c}\left(\eta A_{side}+A_{bot}\right)}$$

where $A_{side}$ represents the total sidewall area of all fins exposed to the coolant, and $A_{bot}$ denotes the total bottom area between the fins, or the “floor” of each microchannel. Given that the geometry of the microchannel fins can significantly influence the convective resistance ($R_{conv}$) [4], optimizing the microchannel fins based on the material used for the spreader can effectively minimize the total thermal resistance ($R_{tot}$) and the temperature rise of the GPU (∆T) under the heat load of Q.

**Figure 2:**
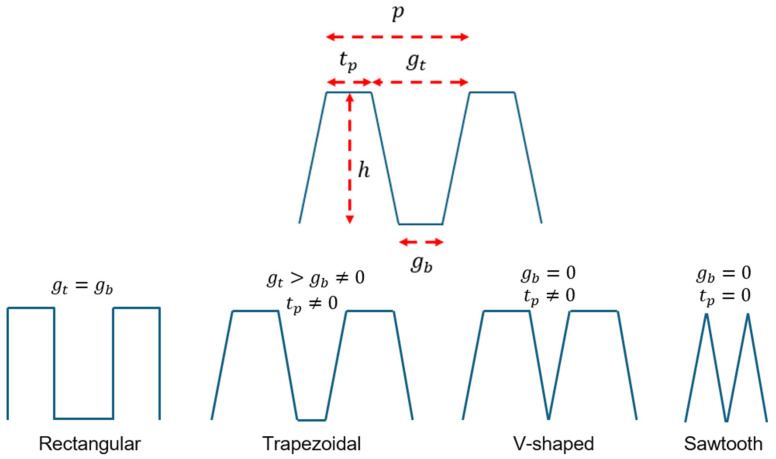
Geometric parameters of the microchannel spreader fins, showing variations in fin shapes, including rectangular, trapezoidal, V-shaped, and sawtooth, based on tip thickness ($t_{p}$), height (h), gaps between adjacent fins at the top ($g_{t}$) and at the bottom ($g_{b}$) and the pitch of the microchannels (p).

The fins of the water-cooled microchannel spreader exhibit a variety of geometries, including rectangular, trapezoidal, V-shaped, and sawtooth, depending on the tip thickness, height, and the gap between adjacent fins at the top and bottom [2,9]. Figure 2 depicts these geometric parameters, as well as the pitch of the microchannels.

As shown in Figure 2, the fins are generally trapezoidal. However, when the gaps at the top and bottom are equal, the fins become rectangular. If the gap at the bottom is reduced to zero, the fins take on a V-shaped form. Finally, if the tip thickness is also reduced to zero, the fins transform into a sawtooth shape.

As seen in Figure 2, the pitch of the microchannels is defined as follows:

(6) $$p=t_{p}+g_{t}$$

Therefore, the fin side length (s) is calculated as follows:

(7) $$s=\sqrt{h^{2}+\;{\left(\frac{g_{t}-\;g_{b}}{2}\right)}^{2}}$$

By knowing the pitch of the microchannels (P) and the fin side length (s), The $A_{side}$ and $A_{bot}$ in Equation (5) can be calculated as follows:

(8) $$A_{side}=\frac{2sA}{P}N_{p}$$

(9) $$A_{bot}=\frac{g_{b}A}{P}N_{p}$$

where $N_{p}$ is the number of fluid passes within the microchannels. The number of channels ($N_{ch}$) is computed from the spreader width $\left(W\right)$ and the pitch (p) of the microchannels as:

(10) $$N_{ch}=int\left(\frac{W}{P}\right)$$

The efficiency of the fins (η) in Equation (5) is expressed in Equation (11) [20]:

(11) $$\eta \;=\frac{\tanh\left(\beta \right)}{\beta }$$

The parameter *β* is defined as in Equations (12) and (13), consistent with classical fin efficiency formulations [3,22].

(12) $$\beta =\sqrt{(\frac{hk_{f}Nu}{k_{s}t_{eq}})\left(\frac{g_{t}+g_{b}+2s}{g_{t}+g_{b}}\right)}$$

where $k_{f}$ is the thermal conductivity of the fluid, and $t_{eq}$ is the equivalent fin thickness which is calculated as follows:

(13) $$t_{eq}=\;t_{p}+\frac{g_{t}-\;g_{b}}{2}$$

The convective heat transfer coefficient or the film coefficient ($h_{c})$ in Equation (5) can be estimated from the Nusselt number (Nu), the conductivity of the fluid ($k_{f}$) and hydraulic diameter ($D_{h}$) as follows [3]:

(14) $$h_{c}=\frac{k_{f}Nu}{D_{h}}$$

The hydraulic diameter ($D_{h}$) of the fins in Equation (14) is a function of the channel area ($A_{c}$) and wetted perimeter ($P_{w}$) which are defined as follows [4]:

(15) $$A_{c}=\frac{h(g_{t}+g_{b})}{2}$$

(16) $$P_{w}=g_{t}+g_{b}+2s$$

Therefore, the hydraulic diameter ($D_{h}$) for the fins can be expressed as follows [4]:

(17) $$D_{h}=\frac{4A_{c}}{P_{w}}=\frac{2h(g_{t}+g_{b})}{g_{t}+g_{b}+2s}$$

The Nusselt number correlation used in this study follows the classical relations for internal flow presented by Incropera and DeWitt (2002) [3]. It is expressed as a function of the Reynolds (Re) and Prandtl (Pr) numbers, covering both laminar and turbulent flow regimes:

(18) $$Nu\left(Pr,Re\right)=\left\{\begin{array}{c} 3.66\;\;\;\;\;\;\;\;\;\;\;\;\;\;\;\;\;\;\;\;\;\;\;\;\;Re<2300\\ 0.023{Re}^{0.8}{Pr}^{0.4}\;\;\;\;\;\;\;Re\geq 2300 \end{array}\right.$$

where the Reynolds (Re) and Prandtl (Pr) numbers are defined as follows:

(19) $$Re=\frac{\rho U_{ch}D_{h}}{\mu }$$

(20) $$Pr=\frac{c_{p}\mu }{k_{f}}$$

where ρ, μ and $c_{p}$ denote the fluid density, dynamic viscosity, and specific heat capacity at constant pressure, respectively.

To ensure smooth continuity between laminar and turbulent regimes, a Laminar–Turbulent Nusselt blending correlation was employed rather than a sharp piecewise transition. The local Nusselt number was computed using a weighted blend of the laminar constant solution ($Nu_{lam}=3.66$) and the Petukhov/Gnielinski (1976) turbulent correlation, with a logistic weighting function w(Re) centered at the critical Reynolds number (Re≈2300) as [21,24]:

(21) $$Nu=[{\left(1-w\right){Nu}_{lam}^{m}+w{\left(\max\left({Nu}_{turb},{Nu}_{lam}\right)\right)}^{m}]}^{1/m}$$

where the weighting function w is the following function of Re number and m = 3.

(22) $$w=\frac{1}{1+exp[-\frac{\left(Re-2300\right)}{400}]}$$

The ${Nu}_{turb}$ in Equation (21) is obtained from the Petukhov–Gnielinski relation as [21,24]:

(23) $$Nu_{turb}=\frac{f/8(Re-1000)Pr}{1+12.7\sqrt{f/8}(Pr^{2/3}-1)}$$

where f is the Petukhov friction factor, which is estimated as follows:

(24) $$f=(0.79ln\;Re-1.64{)}^{-2}$$

This smooth blending eliminates discontinuities in the convective heat transfer coefficient in Equation (14) as $h_{c}=\frac{Nuk_{f}}{D_{h}}$ and improves convergence stability in parametric optimization across the full Reynolds number range.

The mean fluid velocity in each channel ($U_{ch}$) is defined in terms of the volumetric flow rate ($\dot{V}$), the channel cross-sectional area ($A_{c}$), and the number of parallel channels ($N_{ch}$) as follows:

(25) $$U_{ch}=\frac{\dot{V}}{N_{ch}A_{c}}$$

The total pressure drop (∆P) across the microchannel domain was evaluated using a smooth Poiseuille number surrogate that captures the laminar hydraulic behavior of rectangular and tapered ducts. For laminar flow, the pressure loss per channel was estimated as [4,5]:

(26) $$∆P=N_{p}\frac{P_{o}\mu LU_{ch}}{2D_{h}^{2}}$$

where L is the length of the spreader and $P_{o}$ is the Poiseuille number. For a fully developed laminar flow, the Poiseuille number can be estimated as [22]:

(27) $$Po\left(\alpha \right)=24\left(1-1.3553\alpha +1.9467\alpha^{2}-1.7012\alpha^{3}+0.9564\alpha^{4}-0.2537\alpha^{5}\right)$$

where α is a dimensionless number that can be defined as Equation (28).

(28) $$\alpha =\min\left(\frac{\bar{g}}{h},\;\;\frac{h}{\bar{g}}\right)$$

where $\bar{g}$ is the average gap between two adjacent fins or the average width of the microchannels.

(29) $$\bar{g}=\frac{g_{t}+g_{b}}{2}$$

This continuous surrogate ensures numerical stability during optimization while reproducing the correct limiting behavior for both deep ($h\gg g_{t},g_{b}$) and shallow ($h\ll g_{t},g_{b}$) channels. The formulation provides smooth derivatives with respect to geometric variables, which is critical for gradient-based and continuation optimization.

### 2.2. The Optimization Model

The temperature rise of the chip (ΔT) for a given heat load (Q) is minimized by reducing the total thermal resistance ($R_{tot}$) through systematic optimization of the microchannel fin geometry. The optimization spans a multidimensional design space defined by the spreader thermal conductivity ($k_{s}$) from 400 to 2200 W/mK (from copper to CVD diamond), inlet velocity ($U_{in}$) from 0.5 to 5.5 m/s, maximum allowable pressure drop ($\Delta P_{max}$) from 10 to 50 kPa and the number of fluid passes $N_{p}\in \{1,2,3\}$. Within this parameter space, the model determines the geometric configuration that minimizes $R_{tot}\;$ while satisfying manufacturability, geometric integrity, and hydraulic constraints.

The optimization primarily reduces the convective component ($R_{conv}$) through fin geometry adjustment while also capturing the dependence of the base conduction resistance ($R_{base}$) on the spreader conductivity ($k_{s}$). The thermal interface resistance ($R_{TIM}$) remains constant because both the TIM and thickness are fixed across all simulations. The search is conducted within a feasible design region bounded by practical limits on fin height, pitch, tip thickness, and channel gap, subject to system-level constraints including allowable pressure drop and structural integrity. The objective function, defined in Equation (30), minimizes the total thermal resistance as:

(30) $$\underset{h,t_{p},g_{t},g_{b}}{\min}R_{tot}=R_{TIM}+R_{base}+R_{conv}$$

The geometric, hydraulic, and material constraints applied in the optimization model are summarized in Table 1, while the fixed parameters and assumptions used throughout the analysis are listed in Table 2. The geometric and operating bounds in Table 1 and Table 2 were chosen to reflect common manufacturing limits, material conductivities, and pump-driven inlet velocities used in practical microchannel spreaders for high-power electronics.

The optimization problem defined by these constraints and parameters was solved using a deterministic continuation approach, described in Section (c).

### 2.3. Solution Methodology

The optimization problem is formulated within a Lagrangian framework and solved numerically using a penalized merit function, $\Phi \left(x\right)$, that enforces both equality and inequality constraints [25,26]. This approach follows the classical nonlinear programming theory developed by Karush, Kuhn, and Tucker, which defines the necessary conditions for constrained optimality [25,27]. The main computational task involves evaluating the full gradient of $R_{\mathrm{conv}}$, including the derivatives of the heat transfer coefficient and fin efficiency with respect to each design variable. Active constraints, particularly the pressure-drop limit, are incorporated into the stationarity conditions through their corresponding Lagrange multipliers. The design vector is defined as $x=[h,t_{p},g_{t},g_{b}{]}^{T}$, and iterations continue until the gradient norm ∥∇Φ∥ falls below a specified tolerance and all constraints are satisfied. The Lagrangian is expressed as:

(31) $$L\left(x,\lambda ,\mu \right)=R_{conv}\left(x\right)+\sum_{i}\lambda_{i}C_{i}\left(x\right)+\sum_{j}\mu_{j}E_{j}\left(x\right)$$

Since $R_{TIM}$ and $R_{base}(k_{s})$ do not depend on the geometry of the fin, minimizing $R_{tot}$ with respect to x is equivalent to minimizing $R_{conv}$; thus, the Lagrangian in Equation (31) was written with $R_{conv}$ without loss of generality.

In Equation (31), $\lambda_{i}$ and $\mu_{j}$ denote the Lagrange multipliers associated with the inequality and equality constraints, respectively, and $C_{i}(x)$ and $E_{j}(x)$ represent the corresponding constraint functions. The first-order Karush–Kuhn–Tucker (KKT) optimality conditions are summarized as follows:

I. Stationarity
  (32) $$\nabla_{x}L\left(x,\lambda ,\mu \right)=\nabla R_{conv}\left(x\right)+\sum_{i}\lambda_{i}{\nabla C}_{i}\left(x\right)+\sum_{j}\mu_{j}{\nabla E}_{j}\left(x\right)=0$$
  where each gradient component is obtained as:
  (33) $$\frac{\partial R_{tot}}{\partial x_{k}}=\frac{\partial R_{conv}}{\partial x_{k}}=-\frac{\left[\left(\eta A_{side}+A_{bot}\right)\frac{\partial h_{c}}{\partial x_{k}}+h_{c}\left(A_{side}\frac{\partial \eta }{\partial x_{k}}+\eta \frac{\partial A_{side}}{\partial x_{k}}+\frac{\partial A_{bot}}{\partial x_{k}}\right)\right]}{{{[h}_{c}\left(\eta A_{side}+A_{bot}\right)]}^{2}}$$

II. Primal feasibility
  (34) $$C_{i}\left(x\right)\leq 0{,\;\;\;\;\;\;\;\;\;\;E}_{j}\left(x\right)=0$$

III. Dual feasibility

The inequality constraints multipliers must be greater than or equal to zero ($\lambda_{i}\geq 0$)

IV. Complementary slackness

For all inequality constraints, we should have:

(35) $$\lambda_{i}C_{i}\left(x\right)=0\;\;\;\;\;\;\forall i$$

For numerical implementation, the constrained KKT system is converted into an unconstrained form through the penalized merit function, $\Phi \left(x\right)$, as follows [25,26]:

(36) $$\Phi \left(x\right)=R_{conv}\left(x\right)+\rho_{1}\sum_{i}\max{\left(0,C_{i}\left(x\right)\right)}^{2}+\rho_{2}\sum_{j}{E_{j}\left(x\right)}^{2}\;$$

where $\rho_{1},\rho_{2}>0$ are penalty parameters. Large penalty values suppress constraint violations so that, at convergence, $\Phi \left(x\right)$ is minimized and all constraints are satisfied.

The design variables are updated iteratively using a gradient-based rule such as a sequential quadratic-programming (SQP) or quasi-Newton scheme [25]:

(37) $$x^{n+1}=x^{n}-\gamma^{n}\nabla_{x}\Phi \left(x^{n}\right)\;\;\;\;\;\;\;\;\;\;\;\;\;\;\;\;\;\;\;\forall x\in {\left[h,t_{p},g_{t},g_{b}\right]}^{T}$$

where the step size γ is typically between 0.1 and 0.5 to ensure stable convergence. The iterations terminate when we have:

(38) $$∥\nabla_{x}\Phi \left(x^{n}\right)∥\leq \varepsilon$$

with $\varepsilon =10^{-5}–10^{-7}$ representing the gradient-norm tolerance.

The optimization routine was implemented in Python version 3.1 and executed for each combination of spreader conductivity, inlet velocity, number of passes, and allowable pressure drop. The resulting stationary points satisfying the KKT conditions define the optimal fin geometries that minimize $R_{tot}\;$ within the feasible design space established in Table 1 and Table 2.

## 3. Results

The optimization model described in Section (c) was solved for a wide parameter space covering spreader conductivity ($k_{s}=400–2200W/mK$), inlet velocity ($U_{in}=0.5–5m/s$), number of passes ($N_{p}\in \{1,2,3\}$), and maximum pressure drop ($\Delta P_{max}=10–50kPa$). For each operating condition, the optimal geometry vector $(x^{*}=[h^{*},t_{p}^{*},g_{t}^{*},g_{b}^{*}{]}^{T}$) was obtained by minimizing the total thermal resistance $R_{tot}$ within the bounds listed in Table 1. The KKT-based formulation ensures that all geometric and hydraulic constraints are satisfied at convergence.

Under the typical case where the pressure-drop constraint is active, the stationarity condition takes the following form:

(39) $$\nabla_{x}R_{tot}\left(x^{*}\right)+\lambda_{P}\nabla_{x}\left[\Delta P\left(x^{*}\right)-\Delta P_{max}\right]=0$$

where $\lambda_{P}$ is the Lagrange multiplier associated with the pressure constraint, and the maximum pressure drop will be:

(40) $$\Delta P(x^{*})=\frac{Po(\alpha )\mu LU_{ch}}{2D_{h}^{2}}=\Delta P_{max}$$

Solving the KKT system and substituting the equalities from the governing relations yield the closed-form parametric trends for the optimized geometric parameters as reported in Table 3.

Although the simplified parametric expressions in Table 3 provide valuable physical intuition, they treat Nu and Po as weakly varying and therefore cannot fully capture the geometric coupling imposed by the active pressure-drop constraint. In the full KKT system [Equations (39) and (40)], Nu, Po(α), and the hydraulic diameter all depend on the geometry being optimized. Consequently, increasing the spreader conductivity $k_{s}$ reduces the spreading resistance and shifts the balance between conduction and convection in ways not reflected by the simplified scaling relations.

When the pressure constraint is active, the optimizer relaxes extreme aspect ratios as $k_{s}$ increases. This leads to shorter fins, thinner tips, and a slightly more compact pitch, while the flow gaps widen modestly to reduce per-channel losses. The figures that follow quantify these coupled behaviors across the full range of $k_{s}$, $U_{in}$, $N_{p}$ and $\Delta P_{max}$.

**Figure 3:**
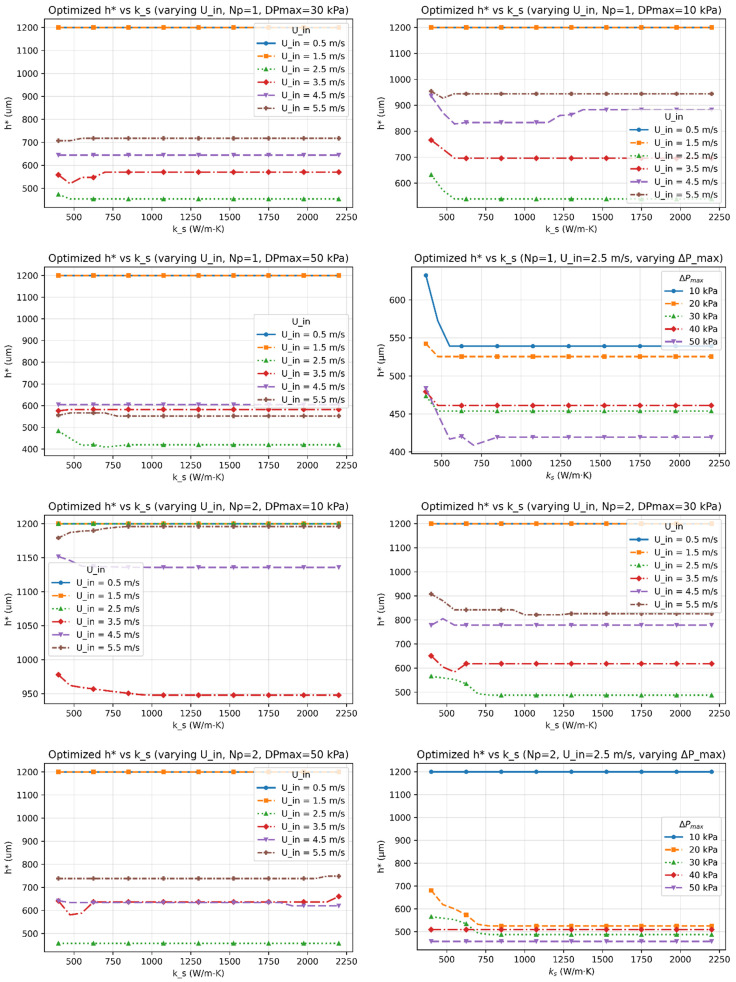

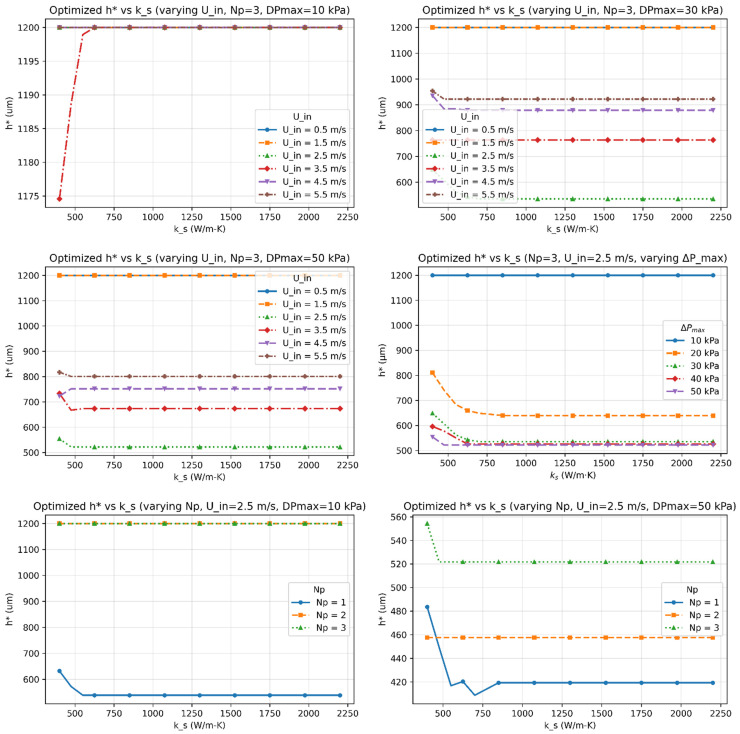
The optimized fin height ($h^{*}$) as functions of the spreader conductivity ($k_{s}$) for multiple inlet velocities ($U_{in}$), number of passes ($N_{p}$), and allowable pressure-drop limits (${∆P}_{max}$).

**Figure 4:**
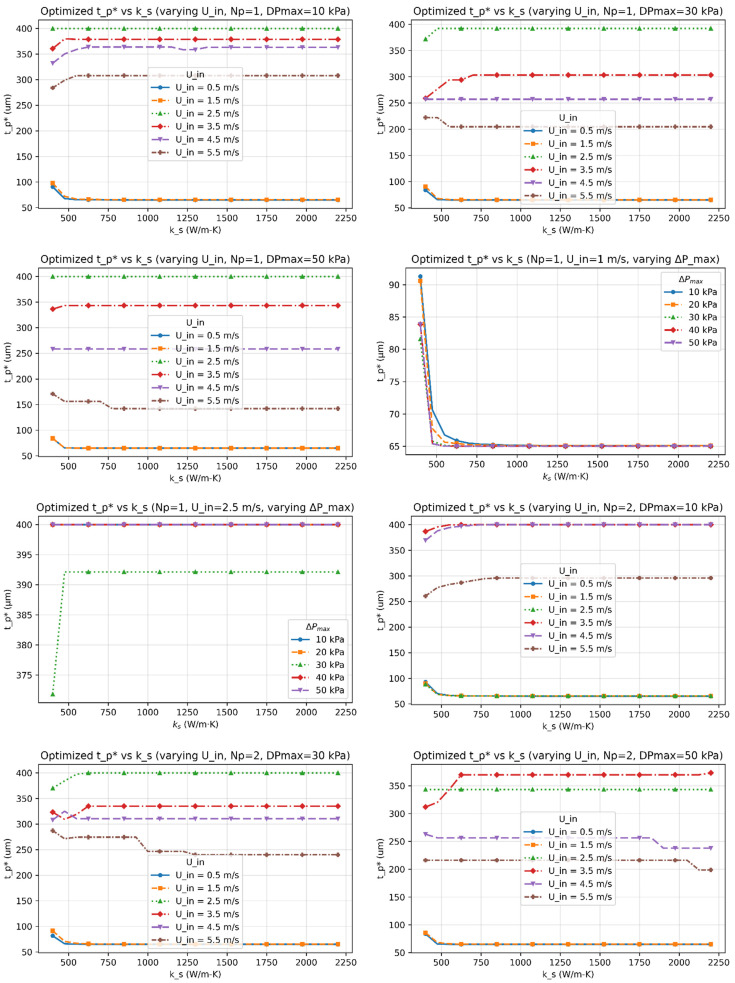

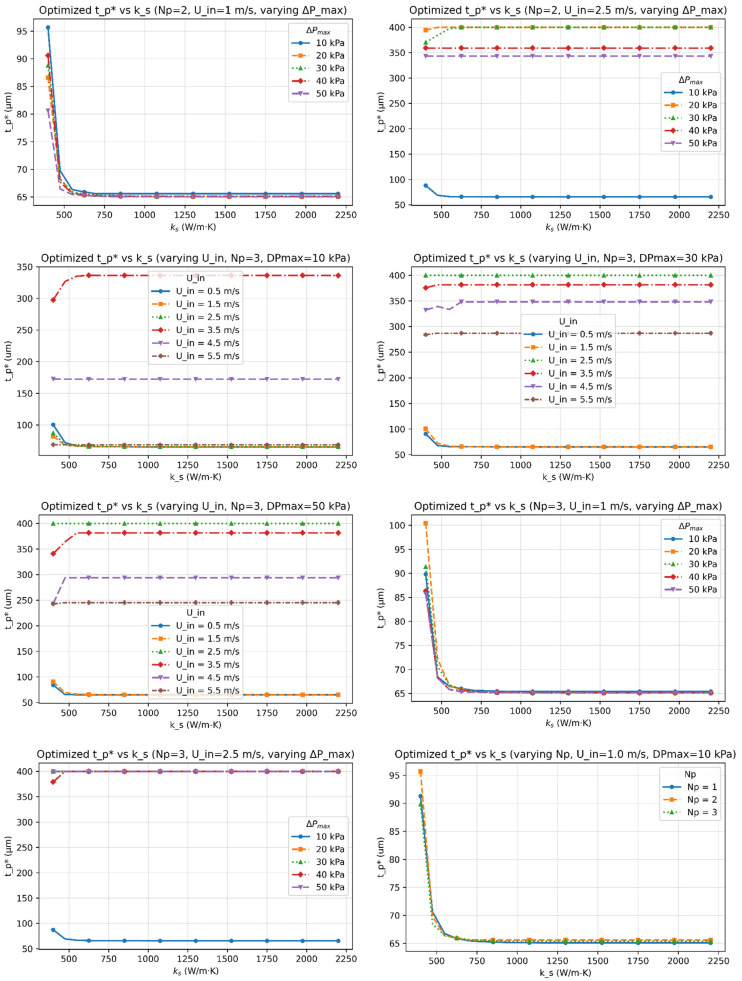

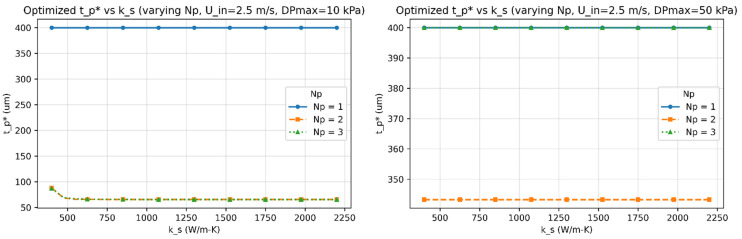
The optimized fin tip thickness ($t_{p}^{*}$) as functions of the spreader conductivity ($k_{s}$) for multiple inlet velocities ($U_{in}$), number of passes ($N_{p}$), and allowable pressure-drop limits (${∆P}_{max}$).

**Figure 5:**
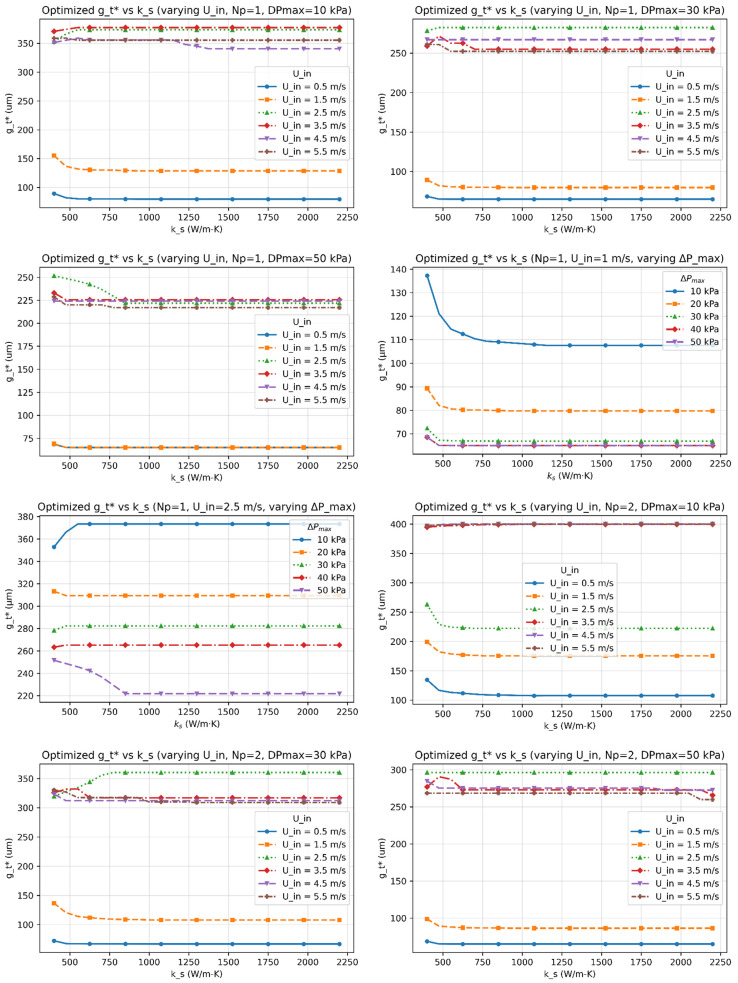

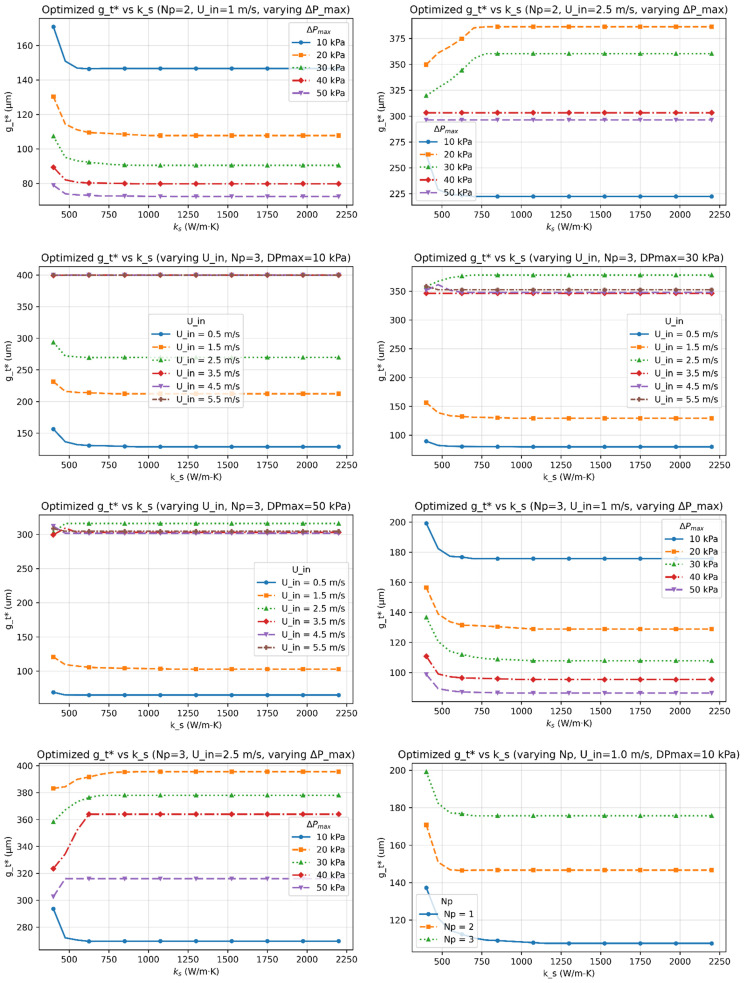

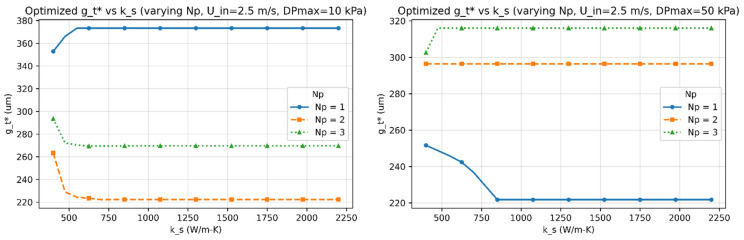
The optimized top gap ($g_{t}^{*}$) as functions of the spreader conductivity ($k_{s}$) for multiple inlet velocities ($U_{in}$), number of passes ($N_{p}$), and allowable pressure-drop limits (${∆P}_{max}$).

**Figure 6:**
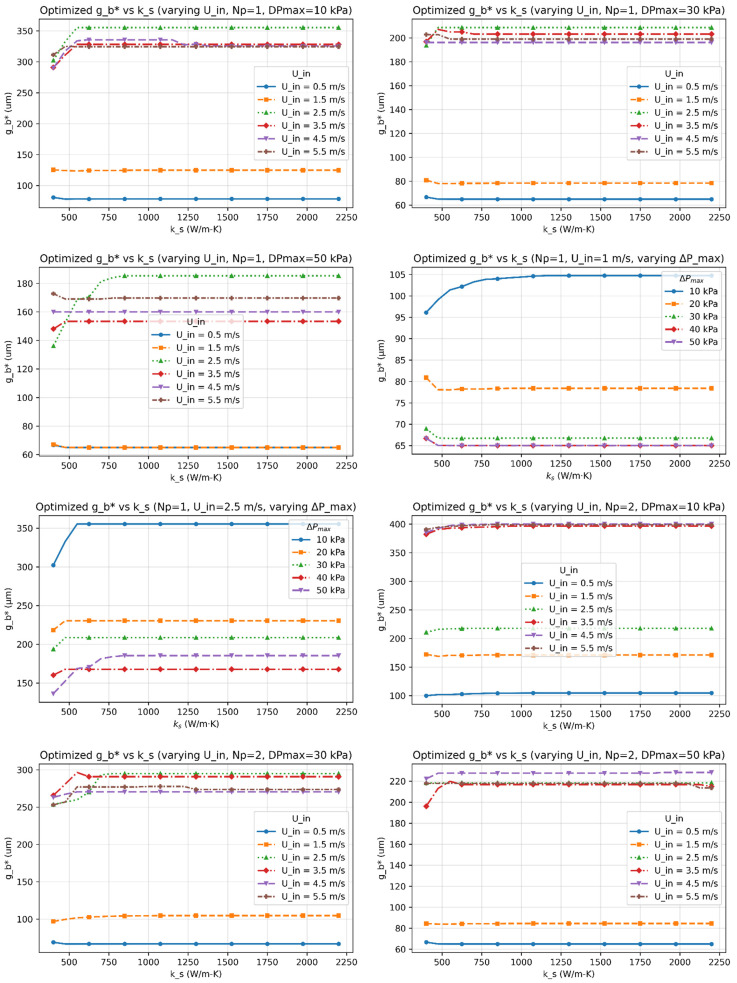

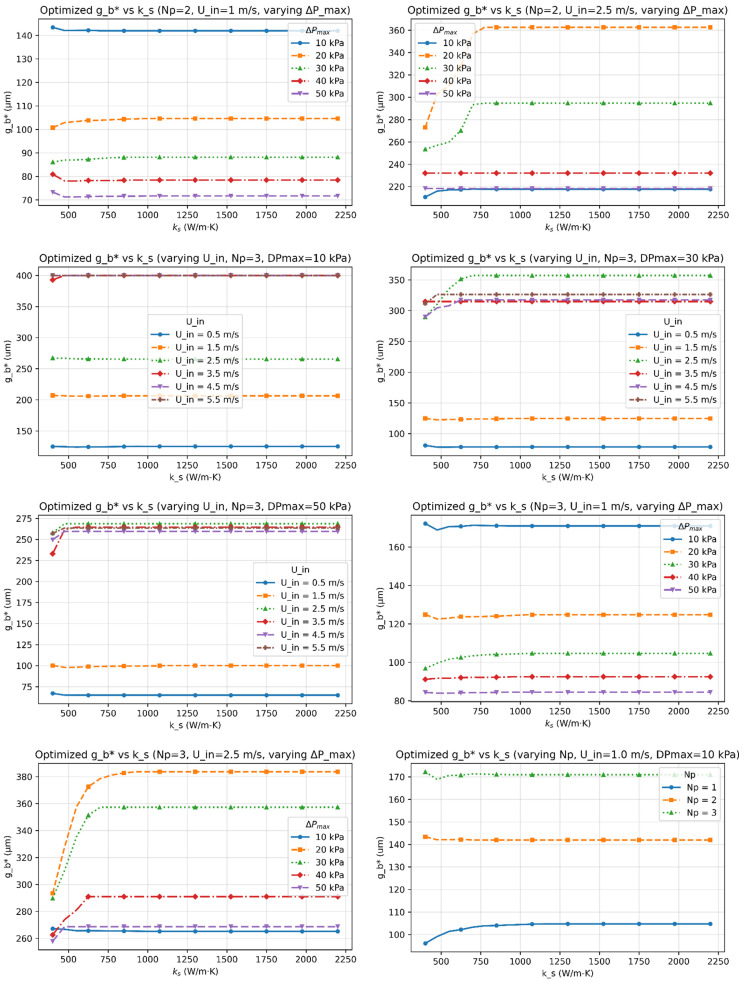

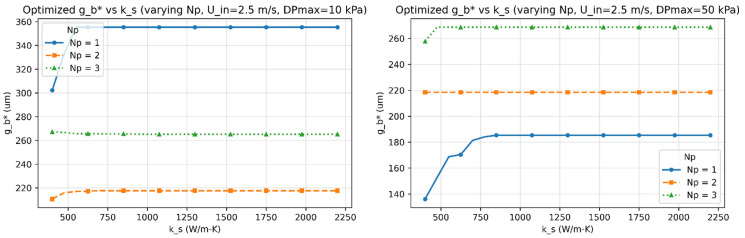
The optimized bottom gap ($g_{b}^{*}$) as functions of the spreader conductivity ($k_{s}$) for multiple inlet velocities ($U_{in}$), number of passes ($N_{p}$), and allowable pressure-drop limits (${∆P}_{max}$).

**Figure 7:**
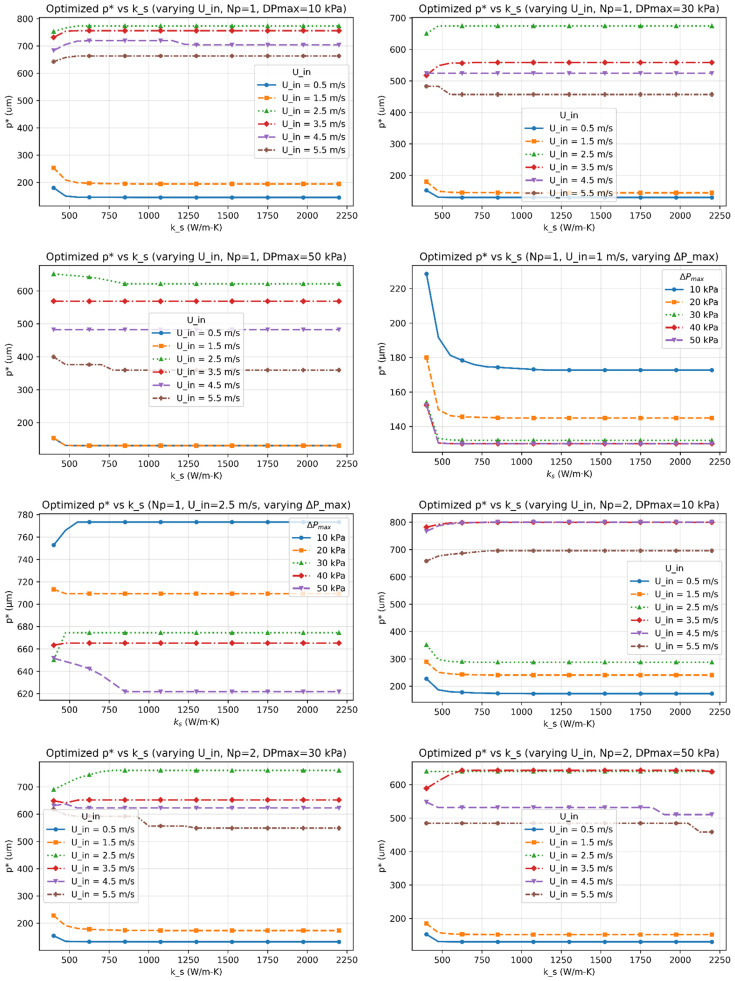

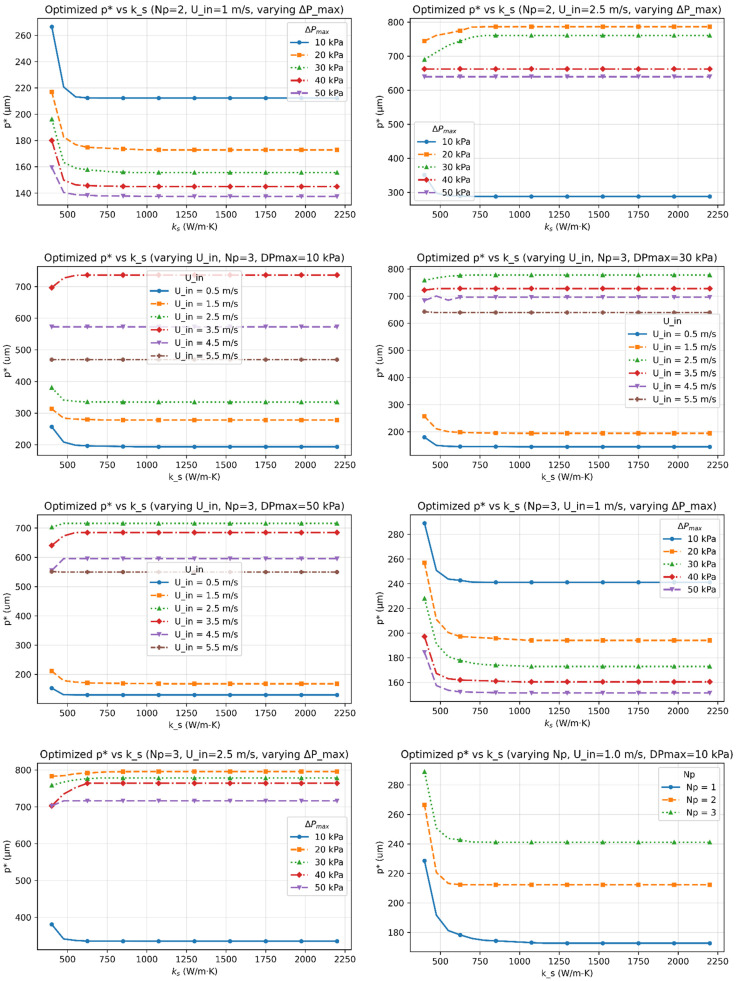

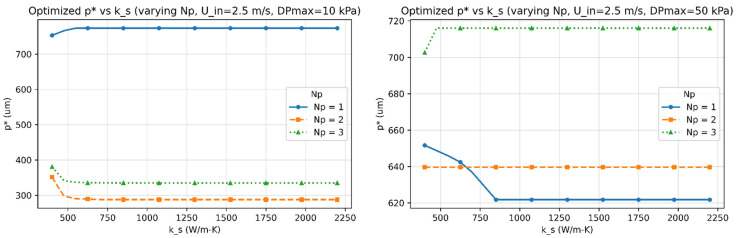
The optimized microchannel pitch ($p^{*}$) as functions of the spreader conductivity ($k_{s}$) for multiple inlet velocities ($U_{in}$), number of passes ($N_{p}$), and allowable pressure-drop limits (${∆P}_{max}$).

Figure 3, Figure 4, Figure 5, Figure 6 and Figure 7 report the optimized geometry vector $x^{*}={\left[h^{*},t_{p}^{*},\;g_{t}^{*},\;g_{b}^{*}\right]}^{T}$ and pitch $p^{*}=\;t_{p}^{*}+g_{t}^{*}$ as functions of spreader conductivity, inlet velocity, number of passes, and allowable pressure drop. Figure 3 presents the optimized fin height h as a function of spreader conductivity for different inlet velocities, pressure-drop limits, and pass counts. Figure 4 shows the corresponding optimized fin tip thickness $t_{p}$ under the same operating conditions. Figure 5 reports the optimized top gap $g_{t}$, while Figure 6 shows the optimized bottom gap $g_{b}$, both highlighting how channel openings adjust with material and hydraulic parameters. Figure 7 summarizes the resulting microchannel pitch p, which reflects the combined influence of the fin dimensions and flow constraints. Together, these plots provide a complete view of how each geometric degree of freedom evolves across the design space. Unless noted otherwise, each solution satisfies the pressure-drop constraint at equality, consistent with Equation (40). The results represent the fully coupled solution of the KKT system, where Nu, Po(α), and the hydraulic parameters co-evolve with the geometry.

When a bound becomes active, for example, when $h^{*}=h_{\max}$ or $t_{p}^{*}=t_{p,\min}$, the corresponding Lagrange multiplier becomes nonzero, and the solution remains fixed at that manufacturing limit.

The following observations summarize how the fully coupled KKT-based optimization modifies the simplified scalings of Table 3 once geometry-dependent convection, conduction, and the active pressure-drop constraint are enforced. It is important to note that the objective function is the minimization of the total thermal resistance $R_{tot}$; therefore, changes in operating conditions do not necessarily require changes in all optimal geometric parameters. In several regimes, different operating conditions converge to identical optimal geometries because $R_{tot}$ becomes insensitive to further geometric variation under active constraints.

I. Effect of Inlet Velocity ($U_{in}$)

As $U_{\mathrm{in}}$ increases, both the Reynolds and Nusselt numbers rise, strengthening convective transport and reducing the convective resistance $R_{\mathrm{conv}}$. In response, the optimizer generally reduces $t_{p}^{*}$, $g_{t}^{*}$, and $g_{b}^{*}$ to satisfy the pressure-drop constraint, leading to a reduced pitch $p^{*}$. However, at low velocities, or when geometric and pressure constraints dominate, different inlet velocities can converge to the same optimal geometry, producing overlapping curves in Figure 3, Figure 4, Figure 5, Figure 6 and Figure 7. Beyond approximately $U_{\mathrm{in}}\approx 3.5–4.5m/s$, further increases in velocity yield diminishing reductions in $R_{tot}$ because the pressure constraint becomes active, limiting additional geometric contraction. This explains why intermediate velocities such as $U_{in}=\;2.5\;m/s$ can occasionally yield smaller optimal dimensions than higher velocities.

II. Effect of Material Conductivity ($k_{s}$)

Increasing $k_{s}$ reduces in-plane spreading resistance, allowing geometric relaxation. This manifests as reductions in $h^{*}$, $t_{p}^{*}$, and $p^{*}$, with modest adjustments in $g_{t}^{*}$ and $g_{b}^{*}$, consistent with Figure 3, Figure 4, Figure 5, Figure 6 and Figure 7. For highly conductive materials, the optimization becomes less sensitive to convective enhancement, and optimal geometries may remain unchanged over a range of operating conditions.

**Figure 8:**
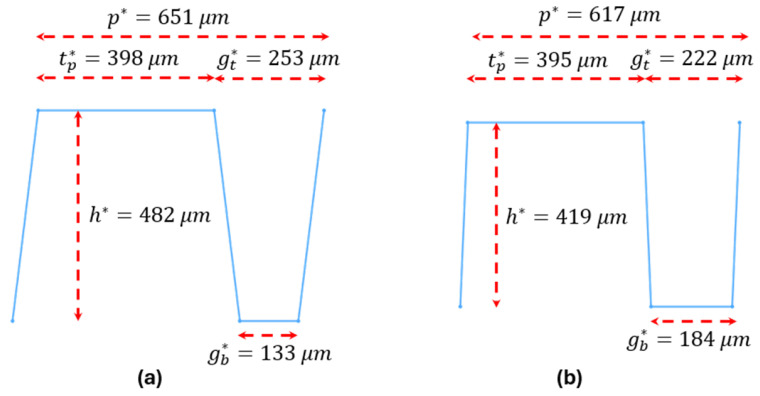
Optimized fin geometries for (**a**) copper and (**b**) diamond microchannel spreaders at $N_{p}=1$, $U_{in}=2.5\;m/s$, $\Delta P_{max}=50\;kPa$ and other parameters as given in Table 2.

To visualize these conductivity-driven changes in the optimized shape of the fins in a microchannel spreader, Figure 8 compares the reconstructed optimized fin geometries for copper and diamond at $N_{p}=1$, $U_{in}=2.5\;m/s$, $\Delta P_{max}=50\;\;kPa$ and other parameters as were defined in Table 2.

III. Effect of Number of Fluid Passes ($N_{p}$)

Increasing $N_{p}$ improves flow uniformity and reduces per-pass mass flux. While this can slightly shorten fins and rebalance gap ratios, the impact on optimal geometry is generally modest. Consequently, some geometric parameters exhibit weak or non-monotonic dependence on $N_{p}$, particularly when $R_{tot}$ is dominated by conduction or constrained by fabrication limits.

IV. Trend of optimized Fin Height ($h^{*}$)

At low velocities or low $k_{s}$, limited convection and weak lateral conduction favor taller fins to enhance heat spreading, causing $h^{*}$ to approach its fabrication ceiling. As $U_{in}$ or $k_{s}$ increases, convective and conductive pathways strengthen, reducing the need for tall fins. In several constrained regimes, $h^{*}$ becomes insensitive to $U_{in}$, leading to overlapping curves that indicate convergence to the same optimal solution.

V. Trend of optimized Tip Thickness ($t_{p}^{*}$)

The optimized tip thickness $t_{p}^{*}$ generally decreases with increasing $k_{s}$ and $U_{in}$. However, this trend is regime dependent rather than universal. In weak flow conditions, when pressure-drop and geometric constraints are simultaneously active, the optimizer may select thin tips even at low $U_{in}$ because $R_{tot}$ becomes insensitive to further increases in $t_{p}^{*}$. Thicker tips are favored primarily when axial conduction limits fin efficiency and the pressure constraint is active. Overlapping curves therefore indicate constraint-dominated optima rather than contradictory behavior.

VI. Trend of optimized Top and Bottom Gaps ($g_{t}^{*}$ and $g_{b}^{*}$)

The optimal gaps are primarily governed by the pressure-drop constraint and geometric consistency ($g_{t}\geq \;g_{b}$). In high-velocity or low-$k_{s}$ regimes, both gaps often approach their manufacturability limits. In contrast, for highly conductive materials or relaxed pressure limits, the gaps widen modestly. Overlapping trends in Figure 5 and Figure 6 reflect regimes where changes in operating conditions do not significantly alter the total thermal resistance.

VII. Trend of Optimized Pitch ($p^{*}$)

The optimized pitch $p^{*}=t_{p}^{*}+g_{t}^{*}$ reflects the combined behavior of $t_{p}^{*}$ and $g_{t}^{*}$. While $p^{*}$ generally decreases with increasing $U_{in}$ and $N_{p}$, non-monotonic behavior can occur in constrained regimes where reductions in $R_{tot}$ are achieved primarily through convective or conductive mechanisms rather than geometric tightening. Thus, increasing $p^{*}$ in certain cases is physically consistent with the global minimization of $R_{tot}$.

## 4. Discussion

The optimization results define a coherent physical and design framework that links material properties, flow conditions, and geometric constraints to overall thermal performance. The observed trends show how each fin parameter evolves to minimize the total thermal resistance ($R_{\mathrm{tot}}$) under coupled conduction and convection limits. Increases in inlet velocity ($U_{\mathrm{in}}$) or allowable pressure drop ($\Delta P_{\max}$) promote compact geometries with thinner fins and narrower channel spacing, thereby enhancing convective transport within the prescribed hydraulic limits. In contrast, higher spreader conductivity ($k_{s}$) and greater pass count ($N_{p}$) allow geometric relaxation, leading to shorter fins and wider channels through improved in-plane heat spreading and reduced reliance on aggressive convective enhancement.

Across the explored parameter space, $R_{\mathrm{tot}}$ decreases monotonically with both $U_{\mathrm{in}}$ and $k_{s}$, although the gains diminish at higher velocities where pumping losses and pressure limits begin to dominate. The transition from copper to diamond consistently yields a 30–45% reduction in $R_{\mathrm{tot}}$, corresponding to several-kelvin decreases in junction temperature under representative chip heat loads. Multi-pass configurations further improve temperature uniformity by redistributing coolant flow and mitigating local hot spots, even when the overall resistance reduction is modest. These findings emphasize that thermal uniformity, in addition to minimum resistance, is critical for reliability in high-power processor cooling.

The interaction between conductivity, hydraulics, and fin geometry forms a continuous design surface: the optimum shifts smoothly from tall, narrow, asymmetric fins for copper and low-flow conditions to shorter, more symmetric fins for diamond and high-flow conditions. This behavior confirms the physical consistency of the optimization model and highlights the intrinsic coupling among material selection, flow configuration, and manufacturable geometry. The derived scaling relations and optimization maps (Table 3) translate these dependencies into actionable design rules, enabling rapid estimation of feasible fin dimensions once the material, flow rate, and pressure budget are specified, without resorting to full computational fluid dynamics or iterative tuning.

Within typical fabrication limits, including minimum feature sizes of approximately 60 µm and maximum fin heights below 1.2 mm, the framework offers a predictive and material-independent methodology for designing liquid-cooled microchannel spreaders across a wide range of conductivities and flow regimes. It thus provides both the physical insight and quantitative foundation needed for the co-design of spreader materials, coolant networks, and pump capacities in next-generation high-power electronic packages.

Although water was used as the baseline coolant, the optimization framework can incorporate any fluid once its thermophysical properties are specified. For example, gallium-based liquid metals such as Galinstan, with their higher thermal conductivity and much higher viscosity, would reduce the convective thermal resistance but substantially increase the pressure drop, shifting the optimal geometry toward wider channels to balance hydraulic losses.

Unlike prior studies that examine individual geometric variations or fixed fin shapes, the present framework provided a unified and shape-agnostic optimization method that simultaneously incorporates conduction, convection, fin efficiency, manufacturability limits, and hydraulic constraints. This integration allows the optimal geometry to emerge directly from the physical and geometric constraints rather than from case-specific assumptions, which distinguishes this work from earlier analytical, numerical, or experimental microchannel investigations.

## 5. Conclusions

This study established a unified optimization framework for minimizing total thermal resistance in liquid-cooled microchannel spreaders used in high-power processor packages. The formulation integrates conduction through the spreader base, interfacial resistance across the thermal interface layer, and convection within parameterized fin geometries, while rigorously enforcing manufacturability and hydraulic constraints through a Lagrangian–penalty approach. By sweeping spreader conductivity ($k_{s}=400–2200Wm^{-1}K^{-1}$), inlet velocity ($U_{in}=0.5–5ms^{-1}$), pressure-drop limits ($\Delta P_{max}=10\;–50kPa$), and pass count ($N_{p}=1$–3), the framework produced smooth, reproducible maps of optimized fins’ geometrical parameters $\left(h^{*},t_{p}^{*},g_{t}^{*},g_{b}^{*}\right)$ across a wide design space.

The optimization results demonstrate that higher inlet velocities or relaxed pressure limits lead to compact fin structures with reduced pitch and enhanced convective efficiency, while higher-conductivity spreaders or multi-pass flow configurations favor shorter, more open channels by mitigating spreading and hydraulic resistances. These findings quantitatively bridge the performance gap between copper and diamond spreaders, enabling up to ~45% reduction in total thermal resistance under equivalent flow conditions.

Beyond its numerical results, the proposed framework offers a practical and material-agnostic design methodology. The resulting correlations and geometry maps allow rapid estimation of manufacturable fin dimensions directly from material and flow specifications, without the need for full CFD modeling. The approach is readily extensible to include anisotropic materials, temperature-dependent properties, or two-phase flow, providing a foundation for the co-design of materials, coolants, and pump architectures in next-generation GPU and power-electronics thermal management systems.

## Figures and Tables

**Table 1:** Constraints and variable bounds applied in fin geometry optimization.

Constraints	Bounds
Maximum pressure drop:	$10\;kPa\;\leq \;\Delta P_{max}\leq \;50\;kPa$
Tip thickness:	$65\;\mu m\leq \;t_{p}\;\leq \;400\;\mu m$
Top gap:	$65\;\mu m\;\leq \;g_{t}\;\leq \;400\;\mu m$
Bottom gap:	$0\leq \;g_{b}\;\leq \;400\;\mu m$
Base fin thickness:	$t_{b}$$=\;t_{p}$$+\;g_{t}$$-\;g_{b}$≥ 65 μm
Fin height:	65 μm ≤ h ≤ 1.2 mm
Geometrical integrity:	$g_{t}\geq g_{b}$
Number of fluid passes:	$N_{p}\in \{1,2,3\}$
Fluid inlet velocity:	$0.5\;m/s\leq \;U_{in}\leq \;5.5\;m/s$
Conductivity of the spreader material:	$400\;W/mK\leq \;k_{s}\leq \;2200\;W/mK$

**Table 2:** Fixed parameters and modeling assumptions for the optimization study.

Parameter	Value
Cooling fluid	Water at 25 °C
Thickness of the TIM layer ($t_{TIM}$)	25 μm
Conductivity of the TIM ($k_{TIM}$)	50 W/mK
Thickness of the spreader base ($t_{base}$)	2 mm
Width of the spreader (W)	30 mm
Length of the spreader (L)	50 mm
Fluid inlet port diameter ($d_{in}$)	5 mm

**Table 3:** Closed-form parametric trends for optimized geometric parameters.

Optimized Parameter	Parametric Trend
Optimum fin height ($h^{*}$):	$\propto {\left(\frac{k_{s}}{k_{f}Nu}\right)}^{1/2}$
Optimum fin tip thickness ($t_{p}^{*}$):	$\propto {\left(\frac{k_{s}}{k_{f}}\right)}^{1/3}$
Optimum top gap ($g_{t}^{*}$):	$\propto {\left[\frac{2D_{h}^{2}\Delta P_{max}}{Po(\alpha )\mu LU_{in}}\right]}^{1/2}$
Optimum bottom gap ($g_{b}^{*}$):	$=g_{t}^{*}-\frac{k_{f}Nu}{2k_{s}}t_{p}^{*}$
Optimum microchannel pitch ($p^{*}$):	$=t_{p}^{*}+g_{t}^{*}$

## Data Availability

The original contributions presented in this study are included in the article. Further inquiries can be directed to the author.
